# Assessment of the half-life of cationic periodontal pocket irrigation

**DOI:** 10.1186/s12903-019-0998-8

**Published:** 2020-01-08

**Authors:** Tugrul Kirtiloglu, Ilker Keskiner, Murathan Sahin, Banu Kirtiloglu, Safak Aygul, Umur Sakallioglu, Gokhan Acikgoz

**Affiliations:** 1 Department of Periodontology, Faculty of Dentistry, University of Ondokuz Mayis, Samsun, Turkey; 2 Department of Nuclear Medicine, Faculty of Medicine, University of Ondokuz Mayis, Samsun, Turkey; 3 Department of Nuclear Medicine, Samsun Training and Research Hospital, Samsun, Turkey; 4 Department of Nuclear Medicine, Medibafra Private Hospital, Bafra, Samsun, Turkey; 5 Department of Periodontology, Faculty of Dentistry, University of Aydin, Istanbul, Turkey

**Keywords:** Cationic irrigation, Periodontal pocket, Scintigraphy, Tallium-201

## Abstract

**Background:**

The concentration and persisting time of antimicrobial agents in the periodontal pockets are important factors for their antimicrobial efficacy. Increased clearance time in the periodontal pocket is a significant criterion for the selection of intrapocket irrigants. The aim of this study was to estimate the clearance time of a cationic agent from the periodontal pocket.

**Methods:**

Thallium-201(Tl-201) was chosen as a tracer to simulate the clearance of cationic substance because of its electrical activity. Twenty patients with periodontitis and probing depths 6 to 9 mm were included in this study. In each patient, 3 Mega Becquerel (MBq) of Tl-201 were inserted into the periodontal pocket. Dynamic imaging was performed and clearance of radioactivity was measured.

**Results:**

Clearance of radioactivity was 67.1 ± 16.9, 83.1 ± 13.9, 90.4 ± 10.4, 93.39 ± 8.0% at 30, 60, 90 and 120 min, respectively. Half-life of wash-out was determined as 20.3 ± 10.2 min.

**Conclusion:**

The results of this study demonstrate that the half-life of the cationic solution applied subgingivally was approximately 20 min and labelling of oral irrigants with radiotracers may be used to determine their clearance in further research.

## B**ackground**

Antimicrobial agents for the support of periodontal treatment are given via rinsing, irrigation, systemic administration, and local application using sustained and controlled delivery devices [1–6].

Oral rinsing is an inefficient method for the introduction of mouth rinses into the periodontal pockets [7, 8]. Therefore, various subgingival irrigation devices (syringe, jet irrigator with a cannula and ultrasonic instrument) have been studied [9–11]. However, Hardy et al. reported that if the irrigating tip was inserted 3 mm subgingivally, complete irrigation was achieved, regardless of pocket depth [12].

Increased clearance time (wash-out from the pocket) in the sulcus is an important aim of selected intrapocket irrigants. According to Oosterwaal et al., crevicular fluid flow affects clearance of the irrigant [13]. Different methods have been reported for evaluation of the effectiveness of drugs used in periodontal treatment. These are the filter paper strip, paper point, fluorometric technique, optical density reading by spectrophotometer and nuclear medicine techniques [13–17].

Some medications bind to the soft and hard tissue walls of the pocket and prolong their clearance time from the periodontal pocket [18]. This property is called substantivity. Some antimicrobial agents show this property because of electrostatic interactions. Chlorhexidine digluconate, which is used as an irrigation and rinsing antimicrobial agent, is a cationic molecule binding to anionic substrates (hydroxyapatite, pellicle, salivary glycoproteins, and mucous membranes) with electrostatic interactions [8, 19]. Due to technical limitations, the evaluation of irrigation substances’ substantivity is difficult in the subgingival region Radioactive tracers facilitate the evaluation of periodontal pocket clearance due to the high sensitivity of small volumes of radiolabelled substance to detection remote from the pocket, and they also allow the measurement of clearance in real time [16].

The aim of the present study was to assess the clearance time of a cationic substantive molecule from the periodontal pocket whether it was slow or not. Tl-201, an easily detectable cationic radioactive agent, was chosen to calculate the clearance time.

## Methods

The research reported in the paper was undertaken in compliance with the Helsinki Declaration and the protocol of this study was previously approved by the Research Ethics Committee of Ondokuz Mayis University (2009/101). The aim of the investigation was fully explained to patients and informed consent was obtained from all patients.

### Study population

Twenty non-smoking patients (5 females and 15 males) aged between 42 and 56 with periodontitis were included in this study. Twenty periodontal pockets (probing pocket depths 6 to 9 mm) belong to single rooted teeth were used. Minimum required sample size was calculated 16 at an alpha value of 0.05. Therefore, 20 periodontal pockets were included in this study.

### Clinical examination

One hour before the study with Tl-201, subgingival curettage was carried out on each periodontal pocket, and only one pocket per subject was used. The margins of the periodontal pockets were sealed with retraction cord to prevent the abrupt overflow of Tl-201 before the dynamic imaging was performed. Retraction cord surrounding the teeth had some space between two ends as small as needle entry. When we did not use retraction cord, most of the oral cavity was contaminated by Tl-201. Therefore, clear image of related area could not be obtained. Four MBq (unit of radiation activity) of Tl-201 was diluted with isotonic solution and three MBq radioactive agent was prepared to obtain better images. Three MBq of Tl-201 in isotonic solution (sterile and non-pyrogenic) was inserted into the periodontal pocket. Firstly, a syringe with a blunted needle of 0.4 mm diameter was placed apically in the pocket and approximately 0,015–0,02 cc radioactive agent injected until it became visible at the margin of the pocket. The retraction cord was then removed and dynamic imaging performed with the patient in the supine position under a gamma camera (GE starcam 4000i XC/T) equipped with a low energy general-purpose collimator. The energy window of the Tl-201 was 72 keV ± 10% and the matrix size was 128 × 128. Acquisition was begun immediately after the insertion of Tl-201 (final molarity strength was 2,02 ± 10^− 8^ mol/L) into the pocket. Clearance of radioactivity from the insertion site was measured at 20-s intervals for 20 min.

### Half-life of cationic pocket irrigation

A quantitative analysis of the kinetic frame was performed for each patient. The region of interest was drawn over the injection site and the time-activity curve was established for each periodontal pocket using mono-exponential curve fitting. The following equation obtained from the curve was used to determine the rate of decline of activity in pockets at 30, 60, 90 and 120 min, and also the half- life of wash-out:

y = m (e^-kt^).

where.

y: the fractional liquid retention at time (t).

m: the constant.

k: the emptying rate in minutes^− 1^.

Pocket depths, clearance at 30, 60, 90 and 120 min and half- life of wash-out were reported as mean ± standard deviation (SD).

## Results

Mean probing pocket depth was 6.85 ± 0.93 mm. Half-life and clearance of 100% of initial radioactivity at 30, 60, 90 and 120 min were calculated from the curves. Clearance of radioactivity was 67.1 ± 16.9, 83.1 ± 13.9, 90.4 ± 10.4, 93.39 ± 8.0% at 30, 60, 90 and 120 min, respectively. Half- life of wash-out was determined as 20.3 ± 10.2 min. Some pockets (patient no 4, 9, 12, 15) have shorter clearance time than other pockets (Table 1).

**Table 1:** Half-life and clearance of radioactivity at 30, 60, 90 and 120 min from periodontal pockets

Patient No	Pocket Depth (mm)	T_½_ (min)	Clearance at 30 min (%)	Clearance at 60 min (%)	Clearance at 90 min (%)	Clearance at 120 min (%)
1	7	30.00	50.70	69.50	81.20	88.30
2	7	35.00	45.80	67.90	81.00	88.70
3	8	24.00	58.70	79.70	90.00	95.10
4	8	8.00	88.70	98.40	99.80	100.00
5	7	15.00	65.40	83.20	91.80	96.00
6	9	40.00	40.40	55.30	66.50	74.90
7	6	14.70	73.60	92.30	97.80	99.40
8	6	25.00	56.20	76.10	87.00	92.90
9	6	4.70	97.40	99.90	100.00	
10	6	20.00	60.70	80.90	90.70	95.50
11	8	17.30	67.30	86.20	94.10	97.50
12	8	8.70	76.60	91.80	97.20	99.00
13	7	20.00	59.50	77.00	86.90	92.50
14	6	36.00	40.60	54.00	64.40	72.50
15	6	7.30	87.10	97.50	99.50	99.90
16	6	20.00	60.80	79.70	89.50	94.60
17	6	21.00	60.80	82.10	91.80	96.30
18	7	11.20	92.20	99.40	100.00	
19	6	32.30	73.10	93.10	98.20	99.50
20	7	16.80	85.90	98.20	99.80	100.00
Mean ± SD	6.85 ± 0.93	20.3 ± 10.2	67.1% ± 16.9	83.1% ± 13.9	90.4% ± 10.4	93.39% ± 8.0

**T**_**½:**_ half-life of clearance

**Fig. 1:**
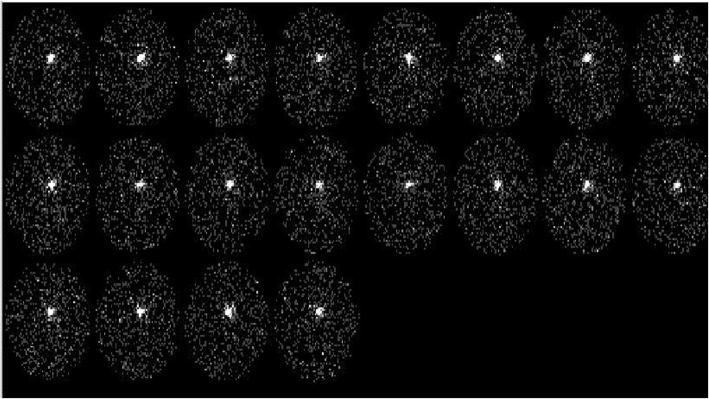
(Patient no. 14) Dynamic images

**Fig. 2:**
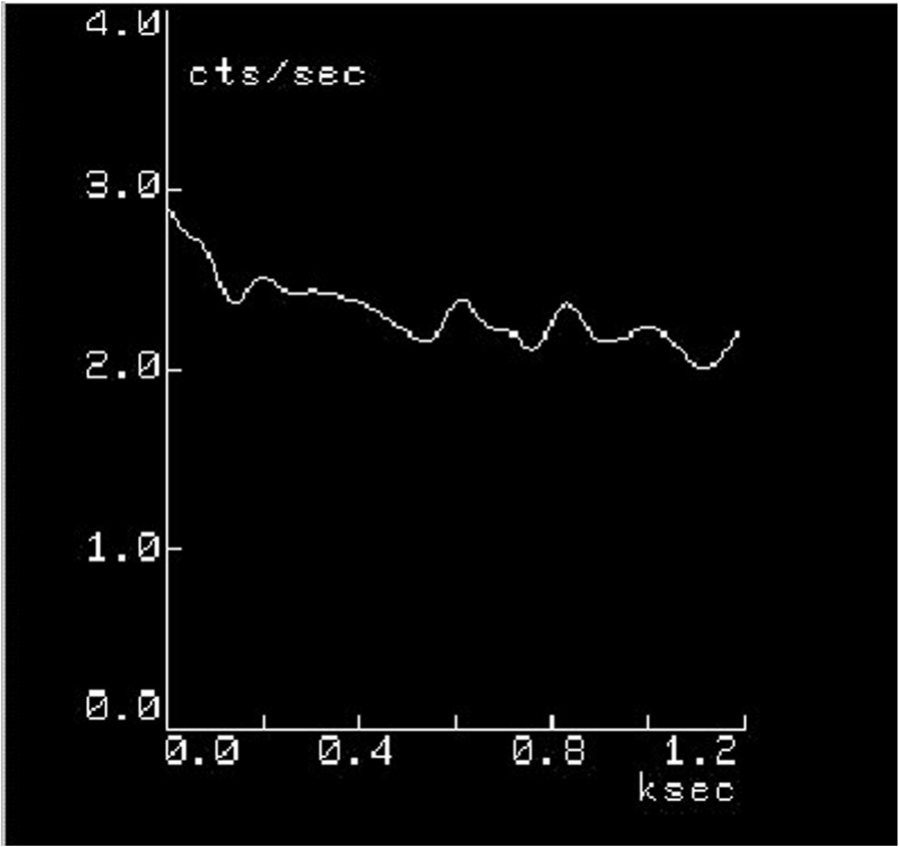
(Patient no. 14) Time-activity curve

Fig. 1 (Patient no. 14) shows 1 min per frame of dynamic images of Tl-201 activity in the periodontal pocket. Time-activity curve obtained from the same patient shows relatively slow wash-out from the pocket (Fig. 2). Scintigraphic images and time-activity curves show decreased activities in the periodontal pockets as a function of time

## Discussion

This study was designed to investigate the clearance time of a cationic substance in real time, but not in the context of therapy. The thallium used in this study is in series IIIA of the periodic table and is readily available as radioactive Tl-201 that has a half life of 73 h. For over 30 years, it has been used in nuclear cardiology studies to assess myocardial perfusion and viability [20, 21]. The current study used Tl-201 to evaluate the clearance of the cationic solution because it is an easily detectable and positively charged substance.

The results of this study demonstrated that most of the cationic substance inserted into the periodontal pocket was washed out within 2 h and some subjects (patient no 4, 9, 12, 15) had shorter clearance time. This could have been caused by periodontal disease severity or by stimulation of fluid flow during tracer insertion and/or subgingival curettage.

The half-life of fluorescein gel persistence in the periodontal pocket was 12.5 min [13] and chitosan persistence using gamma scintigraphy was 41.5 min [16]. In the current study, 80.6% of the radiotracer had cleared from the periodontal pocket at 60 min and the half-life was calculated as 20.3 min. However, this short persistence time could not be enough to achieve a therapeutic concentration. Stimulation of crevicular fluid flow, presence of blood components [7, 22] and dilution of drugs applied in the pocket [23] may limit the effectiveness of subgingival irrigation. A single irrigation of periodontal pockets has limited effects on periodontal healing after scaling and root planning [7]. In contrast, Lander et al. reported that a single subgingival irrigation with 0.2% chlorhexidine digluconate changed the subgingival flora for up to 4 weeks [24].

Goodson estimated that 0.5 μL of periodontal pocket fluid is replaced 40 times/hour [25]. Stabholz et al. reported that subgingival irrigation with 0.12% chlorhexidine digluconate did not exhibit long-lasting substantivity [17]. Oosterwaal et al. reported that when chlorhexidine digluconate gel was applied into the periodontal pocket three times within 10 min, antimicrobial activity could be increased, but this effect continued for only a short time [26, 27].

Subgingival penetration may be limited by the presence of calculus deposits [28, 29]. Hence, subgingival curettage was performed before the application of Tl-201 into the periodontal pockets. However this may have decreased the clearance time because of increasing of gingival crevicular fluid flow.

Boyd et al. inserted the irrigator tip to one-half the depth of the pocket and suggested that the pocket location or the number of roots did not affect the depth of penetration [30]. Single rooted teeth were included in the current study, because we wanted to use approximately similar area and volume of periodontal pockets. Blunt needle was inserted to the base of the pocket and then the substance was injected into the pocket in this study like in the study of Needleman et al. [16].

In this study, only one pocket per subject was used. The reason is that if two or more periodontal pockets were treated simultaneously, the real clearance time of the periodontal pockets could not be calculated exactly due to superposition of activities.

## Conclusion

The results of this study demonstrate that the half-life of cationic solution applied subgingivally is relatively short. In clinical practice, cationic liquid substances applied to the periodontal pocket do not have enough substantivity because of their relatively fast clearance. Further studies could be directed toward improving the substantivity of oral irrigants, and labelling of oral irrigants with radiotracers may used to determine their clearance in further research.

## Data Availability

The datasets used and/or analysed during the current study are available from the corresponding author on reasonable request.

## Abbreviations

Tl-201: Thallium-201
MBq: Mega Becquerel
SD: Standard deviation
